# ETVAX: From Concepts to Demonstrated Clinical Efficacy

**DOI:** 10.3390/microorganisms14071448

**Published:** 2026-06-30

**Authors:** Ann-Mari Svennerholm

**Affiliations:** Department of Microbiology and Immunology, Institute of Biomedicine, Gothenburg University, 45030 Gothenburg, Sweden; ann-mari.svennerholm@microbio.gu.se

**Keywords:** oral vaccines, heat-labile toxin (LT), colonization factors (CFs), mucosal immunity, vaccine efficacy, LMIC children, travelers

## Abstract

This review summarizes the development of an oral inactivated enterotoxigenic *Escherichia coli* (ETEC) vaccine, ETVAX, describing its progression from preclinical evaluation of vaccine components in animal models to clinical assessment of safety, immunogenicity and, ultimately, protective efficacy in first- and second-generation formulations. Multiple Phase 1 and Phase 2 clinical trials conducted, initially in Swedish adults and subsequently in adults and children in Bangladesh and several African countries (Egypt, Zambia and The Gambia), have shown that the candidate vaccines are well tolerated, safe and capable of inducing robust intestinal or intestine-derived immune responses against the included antigens in most vaccine recipients. ETVAX, developed based on the results gained from studies of the first- and second-generation candidate vaccines, comprises four inactivated *E. coli* strains engineered to overexpress the most prevalent colonization factors combined with a toxoid and a mucosal adjuvant. Recent trials have shown that ETVAX confers significant protection against moderate–severe ETEC-associated diarrhea in young children in The Gambia and in travelers to Benin in Africa, underscoring its potential value both for endemic populations and travelers.

## 1. Background

Based on the successful development of an oral cholera vaccine (Dukoral^®^, Valneva Sweden AB, Solna, Sweden) which contains inactivated *Vibrio cholerae* bacteria together with cholera toxin (CT) antigen [1], we explored whether the same concept could be applied to create a vaccine against the cholera-related enterotoxigenic *Escherichia coli* (ETEC) diarrheal disease. Dukoral^®^ was developed following studies demonstrating that *V. cholerae* lipopolysaccharide (LPS) and the B subunit component of CT (CTB) are key protective antigens and that antibodies against LPS and CTB act synergistically to protect against experimental cholera [2]. Studies in mice indicated that peroral immunization with CT was considerably more effective than parenteral administration in inducing intestinal IgA antitoxin responses. The preclinical findings also revealed a close correlation between the magnitude of intestinally synthesized specific IgA antibodies and protection against experimental cholera [3]. Subsequent studies in adult Bangladeshi volunteers confirmed that oral immunization with CTB was superior to parenteral administration in stimulating intestinal immune responses [4]. Importantly the oral cholera vaccine induced comparable intestinal immune responses against bacterial and toxin antigens as clinical cholera [5], which is known to confer long-lasting protection against reinfection.

These findings provided a strong rationale for investigating the development of an oral vaccine against the related ETEC disease. However, ETEC is considerably more heterogenic than *V. cholerae*, and hence, it is more complicated to construct a vaccine with broad coverage against clinical ETEC disease.

Still, there is a great need for an effective ETEC vaccine since ETEC is one of the most common microbial causes of diarrhea in children living in low- and middle-income countries (LMICs) [6,7]. Repeated ETEC infections may also contribute, not only to illness but also to long-term consequences such as stunting, malnutrition and cognitive development [7]. ETEC is also the leading cause of diarrhea in travelers to endemic countries [6,7]. Despite its high global burden there is no ETEC vaccine licensed yet.

## 2. Strategies for Developing an ETEC Vaccine

Based on detailed elucidation of the pathogenic mechanisms of ETEC [6,7,8] which are related to those of cholera, protection against ETEC disease should be directed not only against colonization of the bacteria in the intestine but also against their toxin action. Because immunity in the small intestine is critical for preventing ETEC disease [6,8], strategies to induce strong intestinal immune responses should be explored. Hence, efforts to develop an effective vaccine against ETEC diarrhea have been focused on the identification of major protective antigens inducing antibodies preventing bacterial colonization and capable of neutralizing ETEC toxins and optimal ways of stimulating intestinal immune responses.

## 3. Virulence Factors and Pathogenic Mechanisms of ETEC

At variance with *V. cholerae* bacteria, which only produce one major toxin, cholera toxin (CT), ETEC are very heterogenous bacteria which produce heat-labile toxin (LT), heat-stable toxin (ST) or both toxins (Table 1). These ETEC toxins stimulate fluid and electrolyte secretion in the small intestine, leading to watery diarrhea, and sometimes life-threatening cholera-like diarrhea [7,8]. Immunity against LT is predominantly directed against the B subunit component of LT (LTB) which is 80 per cent homologous to CTB [8]. In contrast, ST is a small peptide of 18 or 19 amino acids and is not immunogenic unless coupled to a carrier protein [9]. Consequently, natural infection with ST-producing ETEC does not induce anti-ST immune responses. The relative distribution of strains producing LT alone, ST alone or LT/ST varies from one geographic area to another and during different times. Globally, approximately 30% of clinical ETEC isolates produce LT only, 30–40% produce LT/ST and 30–40% ST alone [6,10].

ETEC colonize the small intestine by means of proteinaceous colonization factors (CFs), most of which are fimbrial or fibrillar in structure [11]. One of the most prevalent CFs, CS6, is instead an outer membrane protein [11,12]. At least 26 different CFs have been identified to date ([12], Table 1). However, numerous epidemiological studies in different parts of the world have shown that certain CFs are more prevalent than others [11,13,14]. Subsequent studies have also shown that only a few ETEC lineages predominate worldwide and over time [13] and that a few CFs are found on a majority of clinical ETEC isolates [11,14]. Of the wide range of CFs, the most commonly present on clinical isolates include CFA/I and coli surface antigens CS1–CS6, and in some settings CS7, CS14 and CS17 are also common [12,14]. Many ETEC strains express combinations of CFs such as the previously called CFA/II (i.e., CS1 + CS3, CS2 + CS3 or CS3 alone) and CFA/IV (i.e., CS4 + CS6, CS5 + CS6 or CS6 alone), with an increasing number of strains expressing CS6 only [11,12]. The different CFs have been found on ETEC in varying frequencies (50–80%) in different geographic areas, during different seasons and in different categories of patients [11,12,14]. Several CFs belong to related families, i.e., the CFA/I-like group (including CFA/I, CS1, CS2, CS4, CS14, CS17, CS22 and PCFO71) and the coli surface 5-like group (with CS5, CS7, CS18, CS20) [12,15]. Strains expressing CFs within these groups have been shown to induce significant immune responses not only against the homologous strains, but also against other CFs within the respective groups [15,16]. Most CFs consist of more than 1000 identical structural subunits and several of them also express distinct tip proteins [12,15].

ETEC serogroups are similarly diverse, with more than 80 different O antigens identified at variance with *V. cholera* which only have one major Ogroup, O1 ([17], Table 1). In addition, rough strains which lack O antigen are not uncommon [18]. Although some serogroups appear more frequent than others, their distribution varies substantially, making O-antigen-based vaccine strategies impractical. During recent years various noncanonical antigens which may contribute to protection have also been identified [19].

## 4. Studies in Animals

Using a rabbit multiple ileal loop model we demonstrated that antisera against LT as well as purified *E. coli* LPS provided protection against challenge with LT-producing O antigen homologous ETEC strains [20]. Subsequent studies showed that combining anti-LT antibodies with antisera or monoclonal antibodies against different CFs provided significant protection against challenge with LT-producing ETEC strains expressing homologous CF antigens. Furthermore, anti-LT and anti-CF sera acted synergistically to enhance protection against corresponding challenge strains [20]. The protective effects of CFs were supported by experiments in the reversible tie adult rabbit diarrhea (RITARD) model. These studies revealed that intestinal infection with ETEC expressing certain CFs provided highly significant protection against reinfection with fully virulent ETEC expressing the homologous, but not heterologous, ETEC CFs [21]. Using the RITARD model CS6 was shown to function as an intestinal colonization factor and that primary infection with CS6-positive ETEC bacteria protected against disease upon rechallenge with virulent, heterologous CS6-positive ETEC [22].

## 5. Studies in Humans

Evidence for a CF-mediated protective effect of ETEC in humans was indicated by findings from a birth cohort study in the Mirpur urban slum of Dhaka, Bangladesh [23]. In this study 350 children were followed for ETEC infections during their first two years of life. Repeated ETEC infections were extremely common, with some children experiencing up to twenty asymptomatic or symptomatic ETEC infections during the two-year study period. However, reinfections with ETEC expressing the same CF as the initial infecting ETEC strain were rare or absent [23], whereas reinfections with ETEC expressing heterologous CFs were common. These findings strongly support a protective role for CF-specific immunity [23]. Additional evidence comes from human volunteer studies showing that ingestion of bovine milk containing high levels of anti-CF antibodies protected against challenge with virulent ETEC expressing homologous CFs [24].

## 6. Different Candidates of Oral ETEC Vaccines

The overall aim has been to develop an oral inactivated ETEC vaccine capable of providing significant protection against moderate-to-severe ETEC diarrhea in children living in LMICs as well as in travelers to ETEC-endemic countries. The different candidate vaccines developed to achieve this goal are summarized in Table 2.

## 7. The First-Generation ETEC Vaccines

The initial approach to develop an ETEC vaccine focused on creating a prototype formulation consisting of killed *E. coli* bacteria that expressed some of the most common CFs in high quantities and immunogenic form on the bacterial surface [25]. Treatment of CF-positive bacteria with mild formalin (0.5%) inactivated the bacteria without significantly reducing the antigenicity of the fimbrial CFs. Importantly, CFs on the formalin-inactivated bacteria remained stable during long-term storage and even during incubation in gastric juice, at variance with isolated CF fimbriae which are susceptible to proteolytic degradation in the human gastrointestinal tract [25]. Because protection against ETEC has been shown to be enhanced by the synergistic cooperation between CFs and LT, the inactivated CF-positive bacteria were combined with an appropriate LT toxoid. The strong similarity between the B subunits of CT and LT with regard structure, function and immunogenicity [8] and clinical evidence showing that the cholera vaccine containing CTB (Dukoral^®^) has provided 50–70% protection against LT ETEC [26], led to the inclusion of CTB in the initial prototype and first-generation oral ETEC vaccines.

For use in clinical trials it was recommended that CTB should be replaced by recombinantly produced CTB (rCTB) as a toxoid component [27]. Extensive efforts to develop an ST toxoid by recombinant technology [28] or protein synthesis [29] and coupling such molecules to a carrier protein, e.g., CTB, were unsuccessful due to problems producing an immunogenic but nontoxic ST-protein conjugate [9,28]. Based on experience with oral cholera vaccines the ETEC vaccine was also designed for oral administration to induce optimal intestinal immune responses [3,4].

Based on these considerations, a first-generation oral ETEC vaccine was developed in collaboration with SBL Vaccin, Stockholm, Sweden [30]. The formulation consisted of a combination of rCTB and five formalin-inactivated ETEC clinical isolates expressing CFA/I and CS1–CS6 as well as some of the most prevalent ETEC O antigens [30]. However, one of the most common CFs, i.e., the nonfimbrial CS6 supposed to be included in the formulation, did not retain immunogenicity after formalin treatment [30]. The vaccine was planned to be administered in bicarbonate buffer solution as a drink. Thus, previous studies with oral cholera vaccine containing CTB have revealed that in particular the B subunit component is sensitive to gastric acidity in the stomach.

This rCTB-CF ETEC vaccine was initially evaluated for safety and immunogenicity in different studies in adult Swedish volunteers as a basis for continued studies in different populations and age groups. Across all studies the vaccine was safe with no significant adverse events reported among any of the vaccinees [20,30].

Because intestinal immunity is considered of prime importance for protection against ETEC, several different assays were used to evaluate mucosal immune responses in the vaccinated volunteers. In the studies in adult Swedes, anti-CTB and anti-CF IgA antibody responses against the first-generation oral inactivated rCTB-CF ETEC vaccine were determined by the “golden standard” intestinal lavage method [20]. These responses were compared with corresponding immune responses in fecal extracts and serum [20] as well as with intestine-derived IgA antibody-secreting cell (ASC) responses in peripheral blood [30]. Two doses of vaccine given two weeks apart induced significant IgA responses against the various CFs in lavage fluid and feces in most of the vaccinees [20]. Subsequent studies also revealed high frequencies of CF-specific IgA ASC responses (Table 3). Mucosal IgA responses against CTB were observed in 90–100% of the vaccinees as determined by all assays. Similarly, 90–100% of the vaccinees developed serum IgA and IgG responses to CTB, whereas antibody responses in serum against the CFs were mostly infrequent [30].

The frequencies and magnitudes of antibody responses against the CFs and CTB in intestinal lavages correlated significantly with those measured in fecal samples as well as with ASC response [20,30]. Later studies demonstrated that immune responses determined in antibody lymphocyte secretions (ALSs), i.e., in cultures of peripheral blood mononuclear cells (PBMCs), reflected both ASC responses and secretory IgA (SIgA) responses in fecal extracts [31]. Consequently, most subsequent evaluations of mucosal immune responses against candidate ETEC vaccines have been assessed by ALS IgA and fecal SIgA responses against the key vaccine antigens.

Encouraged by the promising results from the studies of the rCTB-CF vaccine in Swedish adults, Phase I and II trials of the vaccine were initiated in Bangladesh and Egypt. In Bangladeshi adults, two oral doses of the rCTB-CF ETEC were safe and well tolerated and induced mucosal immune responses against the different vaccine CFs in 70–100% of the vaccinees [32]. Clinical trials in 200 Egyptian children aged 2–5 and 6–12 years and in infants 6–18 months similarly confirmed that the vaccine was well tolerated with no significant difference in symptoms between vaccine and placebo groups [33,34]. Most vaccinated children developed significant IgA ASC responses against the CFs (83–100%) as well as IgA and IgG antibody responses in plasma against CTB (in 81–93%) with no notable differences between the age groups [33,34]. Comparable frequencies of ASC responses against the CFs (73–87%) and CTB (90%) were observed in 30 Bangladeshi children aged 18–36 months who received two oral doses of the vaccine [35]. Although the vaccine was safe in these children [35], Bangladeshi infants aged 6–17 months vomited when given a full dose of the vaccine [36]. Subsequent dose-finding studies showed that a quarter dose was safe also in infants and elicited significant immune responses against the CFs and LTB [36].

The vaccine efficacy (VE) of the rCTB-CF ETEC vaccine was evaluated in two large placebo-controlled Phase III trials in American travelers going to Mexico and Guatemala [37,38]. The first study involving nearly 700 volunteers receiving two doses of vaccine or placebo two weeks apart, did not meet the primary endpoints of preventing all ETEC cases, including mild cases. However, the vaccine provided significant protection (VE = 77%; *p* = 0.039) against non-mild ETEC diarrheal illness, defined as symptoms interfering with daily activities [37], while no significant protection was observed against ETEC diarrhea of any severity. In a similarly sized follow-up study using non-supervised vaccination, a VE of 60% (*p* = 0.1) was observed against moderate–severe disease caused by ETEC strains expressing vaccine-shared antigens. However, among volunteers who mounted immune responses against CTB, supporting vaccine “take”, efficacy was highly significant (VE = 84%; *p* < 0.01) [38].

A placebo-controlled trial in rural Egypt evaluated the vaccine in 350 children aged 6–18 months After two doses, children were followed by active surveillance, with semi-weekly household visits and fecal sampling from children with diarrhea [39,40]. However, no significant protection was observed (VE = 20%). This limited efficacy may partly reflect the active surveillance approach which is known to detect predominantly mild cases and result in lower VE estimates, as compared with passive surveillance focusing on more severe disease [39,40].

## 8. Further Development of the rCTB-CF ETEC Vaccine

The lack of significant protection in Egyptian children resulted in efforts to improve vaccine efficacy. These efforts focused on increasing the expression of protective antigens, especially the CF antigens on the bacterial surface, without increasing the number of bacteria [39]. Using recombinant technology, CF antigens were expressed in considerably higher quantities on the engineered *E. coli* strains than on the clinical ETEC isolates used in the first-generation vaccine [41]. This was achieved by placing CF genes behind strong promotors, e.g., *tac*, on plasmids into nonvirulent *E.coli* K12 bacteria or a toxin negative O78 ETEC strain. The resulting recombinant *E. coli* strains expressed up to ten-fold higher levels of fimbrial CF antigens compared with the original ETEC vaccine strains. CF expression was determined using monoclonal antibody-based inhibition ELISA methods and immunoelectron microscopy [41]. Special emphasis was applied on overexpressing the nonfimbrial CS6 antigen, which had been non-immunogenic in the first-generation ETEC vaccine. Using *thyA* as a non-antibiotic-based selection marker, a nontoxigenic *E. coli* strain was constructed by electroporating a CS6 *thyA* plasmid into a C600 ∆*thyA* strain. This recombinant strain expressed up to 20-fold higher quantities of CS6 compared to previously tested natural clinical isolates [42]. Because CS6 was found to be sensitive to formalin, inactivation with retained immunogenicity was successfully achieved by phenol treatment of the recombinant CS6-expressing *E.coli* strain [42].

Additional strategies to enhance the efficacy of the rCTB-CF ETEC vaccines included the addition of a toxoid more closely resembling LT, specifically a hybrid LTB/CTB (LCTBA) toxoid [43]. This hybrid protein was shown to be safe and elicit stronger LT neutralizing immune responses than CTB, both in experimental animals [43] and in subsequent human studies [31]. The efforts also involved evaluating various mucosal adjuvants, particularly the double-mutated LT (dmLT) molecule [44], for their potential to enhance vaccine-induced immune responses. Initial studies indicated that dmLT was safe in animals and humans and exhibited strong adjuvant activity by significantly improving the immunogenicity of the rCTB-CF ETEC vaccine in experimental animals [45]. Furthermore, dmLT significantly enhanced CS6-specific mucosal immune responses against the ETEC vaccine in humans [31].

## 9. A Prototype Second-Generation ETEC Vaccine

To evaluate improvements over the first-generation ETEC vaccine, a prototype vaccine containing killed recombinant *E. coli* bacteria overexpressing CFA/I on the bacterial surface combined with the recombinant LCTBA protein, was developed as a model for a second-generation ETEC vaccine. This prototype vaccine was evaluated in a randomized, double-blind phase I trial involving 60 adult Swedish volunteers [46]. The safety and immunogenicity of the prototype vaccine were compared to the reference first-generation rCTB-CF ETEC vaccine in volunteers given two oral doses of either vaccine. The prototype vaccine was administered at the same or a fourfold higher dosage of the CFA/I component and the toxoid compared to the reference vaccine. No significant difference in safety was observed between the prototype at either dosage and the reference vaccine. Most volunteers immunized with the prototype developed fecal SIgA antibody responses against LTB which were slightly higher than those induced by rCTB-CF ETEC vaccine and significantly higher and more frequent in the higher dose group [46]. Immunizations also induced elevated fecal SIgA antibody responses against CFA/I which were somewhat higher in the prototype groups than in the reference vaccine group. The prototype vaccine induced ALS responses against LTB in 95–100% of all vaccinees and in 70–79% against CFA/I of those receiving the higher dose [46].

## 10. The Second-Generation Multivalent ETEC Vaccine

Based on the promising results from the study of the prototype vaccine a more comprehensive multivalent ETEC vaccine was developed comprising inactivated CF-positive ETEC strains expressing 4–10 times higher levels of the CFs than those expressed in the rCTB-CF ETEC vaccine [41,42]. This second-generation vaccine was developed in collaboration with Scandinavian Biopharma, Sweden, using candidate strains optimized for large-scale production under good manufacturing practice (GMP) conditions. The vaccine included three strains overexpressing CFA/I, CS3 and CS5, respectively, with an *E. coli* O78 as the host strain and a fourth strain overexpressing CS6 using *E. coli* K12 as host strain [45,46]. The vaccine preparation, combining CF-overexpressing bacteria with the LCTBA toxoid, was planned to be tested with or without dmLT adjuvant in subsequent studies.

In a preclinical study in mice this second-generation LCTBA-CF ETEC vaccine elicited strong mucosal and systemic immune responses against LTB and each of the vaccine CFs, which were significantly enhanced by coadministration with dmLT adjuvant [46]. These findings supported subsequent human trials to evaluate safety and immunogenicity enhancement by the adjuvant.

A clinical trial in adult Swedish volunteers assessed the safety and immunogenicity of the LCTBA-CF ETEC vaccine with or without dmLT adjuvant [31]. The study specifically analyzed the capacity of the vaccine to induce mucosal, i.e., SIgA immune responses in feces and intestine-derived ALS and/or ASC IgA, responses against the different vaccine antigens. Four equally sized groups of altogether 129 adult Swedish volunteers received two doses two weeks apart of vaccine alone, or together with 10 µg or 25 µg of dmLT adjuvant, or placebo.

The vaccine demonstrated a favorable safety profile with no significant differences in adverse events in the vaccinees compared to in the placebos. Mucosal immune responses against all the CFs, LTB and O78 LPS were induced in most volunteers, 70–80% of the volunteers in ALS and 60–70% in fecal specimens, in the three vaccine groups (Table 4). The addition of 10 µg dmLT significantly enhanced the magnitude of the immune response against the weakest antigen, i.e., CS6. Strong and frequent IgA as well as IgG antibody responses against LTB were induced in 97% and against O78 LPS in 77% of the vaccinees, while serum immune responses against the different CFs were rare or absent [46].

The potential of the vaccine to induce immune responses against antigens not included in the formulation was tested using mucosal samples from the vaccinated Swedish volunteers. Strong and frequent immune responses were observed both in fecal and ALS samples against immunologically cross-reactive CFs, i.e., against CS14 and CS17 which are related to CFA/1, and CS7 which is related to CS5 [16].

To evaluate whether the primary immunizations with the LCTBA-CF vaccine had induced an immunological memory, participants from the three previously vaccinated groups were followed up one to two years later and administered a single booster dose of the vaccine without dmLT. Immune responses against this late booster in the previously immunized individuals were compared with responses to a single vaccine dose in naïve subjects (previous placebos or newly recruited individuals) [47]. The previously immunized volunteers [47].exhibited strong mucosal immune responses against all vaccine antigens following the single booster vaccination, whereas no or weak such responses were induced to the single dose in the naïve subjects [47]. Volunteers initially given the vaccine alone mounted comparable immune responses to the single vaccine dose as those primed with vaccine plus dmLT [47]. These findings indicate that the vaccine induced immunological memory lasting for at least 18 months. The duration of memory may be considerably longer as earlier studies of the oral cholera vaccine Dukoral^®^ demonstrated memory responses against the CTB component of the vaccine persisting for more than 10 years [48].

## 11. Clinical Trials of ETVAC in Children and Adults in LMICs

### 11.1. Studies in Bangladesh

Based on the promising results in the Swedish adults, a large, dose-escalation, age-descending phase 1/2 trial of the LTCBA-CF vaccine was undertaken at icddrb in Bangladesh. Initially the safety and immunogenicity of the vaccine was analyzed in 45 adults given two doses of either the vaccine alone or together with 10 µg dmLT, or placebo [49]. The results showed that the vaccine was safe and well tolerated and induced ALS responses against all five primary vaccine antigens in 100% and strong plasma immune responses in 60–100% of the vaccinees.

Following these encouraging findings in the adults, a pediatric study of safety and immunogenicity of the vaccine was initiated in descending age groups of children in Bangladesh to determine the highest tolerable dose of the LCTB-CF vaccine, with or without dmLT adjuvant [50]. The studies also included evaluation of mucosal and systemic immune responses against the five primary vaccine antigens, i.e., CFA/I, CS3, CS5, CS6 and LT [51]. A total of 450 children were enrolled and randomly assigned to three age groups (120 aged 24–59 months, 100 aged 12–23 months and 230 aged 6–11 months). The participants were given two doses of vaccine (1/2, 1/4 or 1/8 of a full dose), alone or together with different doses (2.5, 5 or 10 µg) of dmLT adjuvant, or placebo [50].

No solicited adverse events occurred that were greater than moderate in severity, and most were mild. The most common adverse event was vomiting which was observed in 8–15% of the children, mostly of mild severity and related to dose and age. The addition of dmLT did not modify the safety profile. Mucosal IgA antibody responses in ALS specimens were detected against all primary vaccine antigens in the two older age groups (Table 5), whereas ALS responses were less frequent in the 6–11-month-old infants. However, fecal SIgA immune responses against all vaccine antigens were recorded in the infants (Table 5) [50].

Interestingly, whereas the mucosal immune response against the different CFs decreased with age among the ETEC vaccinees, corresponding immune responses increased with decreasing age in the nonvaccinated placebo group (Table 5). This finding in the placebo group supports previous findings of frequent ETEC infections during the first years of life in high endemic settings such as Bangladesh [23].

The trial further demonstrated that one-quarter of a full adult vaccine dose was safe and immunogenic even in the youngest age groups, and that dmLT enhanced the magnitude, breadth and kinetics of immune responses in infants [50]. The impact of dmLT on anti-O78 LPS immune responses and whether such responses can predict responses against the CFs as markers for vaccine “take” were also evaluated. The results showed that the fecal immune responses against O78LPS were comparable to those elicited against the CFs in infants, and that dmLT significantly enhanced O78LPS immune responses in this age group [51].

### 11.2. Studies in Zambia

To further evaluate the safety and immunogenicity of the LCTBA-CF ETEC vaccine in young children and infants in LMICs, a Phase 1 trial was undertaken in Zambia [52]. The primary objective was to identify the highest safe and immunogenic dose for use in children in future studies of ETVAX^®^ (Etvax AB, Solna, Sweden). For this study 146 infants aged 6–9 months and 60 young children aged 10–23 months received three doses of vaccine or placebo on days 1, 14 and 90 of either one-quarter or one-eighth of a full adult dose, each co-administered with 2.5 µg dmLT, or placebo. Following each immunization children were monitored for adverse events, both solicited and unsolicited, for 7 days after each dose through home visits by experienced health workers. Immune responses against the different vaccine antigens were analyzed in plasma samples collected immediately before vaccination and then 7 days after the second and third doses [52].

Before immunizing the children 30 adults received a single full dose of ETVAX and 10 adults received a placebo to ascertain safety. This analysis did not reveal any difference in the frequency of solicited adverse events between the vaccine and placebo recipients. The study in children confirmed that the vaccine was safe and tolerable and that most of the solicited adverse events were mild or moderate in both age groups. Unlike the findings in children in Bangladesh, overall vomiting did not differ significantly between the vaccine and placebo groups in Zambia, although vomiting was slightly more frequent after the one-quarter dose than after the placebo in the 6–9-month-old infants (*p* = 0.07) [52].

Determination of plasma immune responses showed statistically significant LTB-specific responses, both with regard to magnitudes and frequencies in 80–95% of the children, with comparable responses after the second and third doses as well as between the one-quarter and one-eighth doses in both age groups. Statistically significant immune response against the CFs was only recorded against the CS3 component in the older children, likely due to elevated baseline anti-CF antibody levels against the other vaccine CFs from prior natural infections. However, infants receiving the one-quarter dose, likely less subjected to natural priming, developed statistically significant immune response, both with regard to magnitudes (fold-rise in vaccinees compared to placebos) and frequencies against CFA/I, CS3 and CS5 after the third dose and significant IgA as well as IgG responses against LTB both after the second and third dose [52]. Based on these results, the one-quarter dose was recommended for evaluation of the protective efficacy of ETVAX in young children in LMICs.

## 12. Evaluation of Protective Efficacy of ETVAX in Children in the Gambia

Based on the results from the studies of children in Bangladesh and Zambia, a large Phase 2b trial was conducted in The Gambia to assess the protective efficacy of ETVAX in young children [53,54]. In this double-blind, placebo-controlled trial, 4936 Gambian children aged 6–18 months were randomized to receive three doses of either a quarter of an adult dose of ETVAX or placebo on days 1, 15 and 90. After immunizations were completed the first 350 children enrolled were actively followed up by daily home visits for adverse events during 7 days after each immunization, while all children were observed for unsolicited adverse events during the whole study. Children with moderate-to-severe ETEC diarrhea (MSD) were told to visit the local health clinics to provide fecal specimens, collected as rectal swabs, for microbial analyses during the study period of 18 months. Protection was assessed as the number of vaccine-preventable outcomes (VPOs), defined as ETEC strains producing LT alone or in combination with ST and/or expressing one or more of the vaccine CFs in the diarrheal stools. A subset of the children was also evaluated for vaccine immunogenicity [53].

ETVAX was safe and well tolerated. Severe adverse events were observed in 1.0% of the vaccinated children and in 1.3% of the placebo recipients. Overall, adverse event rates were similar between the vaccine and the placebo groups [53,54]. The only adverse event that was somewhat more frequent in the vaccinees than in the placebos was vomiting within 30 min after administration.

Analysis of the diarrheal specimens was performed using the Novodiag Bacterial GE PCR assay. When ETEC was detected, corresponding fecal samples were cultured for enteric pathogens, i.e., ETEC, *Shigellae*, *Salmonellae* and *Campylobacter.* Isolated ETEC-positive colonies were subsequently tested by PCR, dot blot and GM1-ELISA for identification of toxins and CFs as previously described [55]. Additional PCR screening for other enteric pathogens including, i.e., other diarrhoeagenic *E. coli* and *Aeromonas*, viral (e.g., adenovirus and norovirus) and parasitic pathogens (*Giardia lamblia* and *Cryptosporidium*) was performed by PCR at Synlab in Finland [56]. These analyses revealed that copathogens were highly prevalent among ETEC-positive samples. Thus, as many as 95% of the ETEC-positive MSD cases, (33% from the vaccinees and 67% from the placebos) contained co-infections [54]. The most frequently detected copathogens were *Campylobacter*, *Giardia lamblia* and enteroaggregative *E. coli* (EAECs). Because almost no VPO cases with ETEC only could be identified, it was concluded that vaccine efficacy should be determined for all VPO cases without excluding copathogens.

In the primary efficacy analysis, which only excluded ETEC-positive MSD cases co-infected with Cryptosporidium, norovirus GII, rotavirus or Shigella spp, vaccine efficacy (VE) was 26.6% (ns). However, when using the modified case definition, i.e., without excluding any copathogens, and initially four and later three or more loose or liquid stools and dehydration symptoms in 24 h, a vaccine efficacy (VE) of 50.9% (*p* = 0.023) was recorded. When excluding cases with parasitic infections, i.e., *Giardia lamblia* or *Cryptosporidium*, VE increased to 80.6% (*p* = 0.0092). Vaccine efficacy against ETEC-positive MSD cases was considerably higher for those children who received their first dose between 6 and 9 months of age than for the older children who received the first dose between 10 and 18 months, i.e., VE = 67.8% (*p* = 0.026) vs. VE = 19.0% (ns) [54]. The lower protection in the older children was likely due to older children in LMICs having been immunized by natural ETEC infections as indicated in the studies of immune responses in children in other ETEC-endemic countries, e.g., Bangladesh and Zambia [50,52].

The protective effect induced by the vaccination against all-cause diarrhea, including cases without detectable ETEC, was also evaluated. Interestingly, it showed that ETVAX conferred significant overall efficacy, VE = 21.4% (*p* = 0.032) against all MSD cases, suggesting potential cross-protection against non-ETEC pathogens [54].

Immune responses against ETVAX antigens in serum were evaluated in two subgroups comprising 122 children, one group at the beginning of the study and the other approximately 12 months later, to confirm consistent immunogenicity throughout the study period. The comparison showed comparable immune responses between the two groups [54]. After three immunizations, immune responses against the CFs were significantly higher and more frequent in vaccine recipients than in the placebo group, and were also higher than those after two doses. In contrast, LTB-specific IgA and IgG responses reached maximal levels already after two doses [54].

The results from the trial in The Gambia were recently highlighted by *Scientific American* as a first-of-its-kind vaccine to protect children against severe or deadly intestinal infections [57].

Based on the promising results from the study in The Gambia a phase 3 trial to evaluate the protective effect of ETVAX against MSD ETEC in children aged 6–18 months in two or three LMICs is planned.

## 13. Evaluation of Protective Efficacy of ETVAX in Travelers

Despite the substantial increase in international travel to LMICs in recent years and the fact that as many as 20–50% of travelers develop travelers’ diarrhea (TD) [58], no ETEC vaccines for TD have yet been licensed. Because the oral inactivated LCTBA-CF ETEC vaccine had previously been shown to be strongly immunogenic in Swedish adults [31,48], a clinical trial was designed to investigate the safety, immunogenicity and efficacy of ETVAX in Western travelers. A suitable study population was identified among Finnish adults traveling for short visits to a cultural center in Benin, West Africa [59].

A double-blind, randomized, placebo-controlled Phase 2b trial was conducted in 746 Finnish adults [59,60]. Participants were randomized 1:1 to receive two oral doses of ETVAX or placebo 1 to 4 weeks before travelling to Benin for a 12-day stay. Stool and blood samples were collected before departure and participants recorded stool frequency, consistency, symptoms and medication use daily during their stay in Benin and for 7 days after returning to Finland [59].

The primary objective of the trial was to assess the safety and immunogenicity of ETVAX^®^. The vaccine demonstrated an excellent safety profile consistent with previous studies in adults from different countries with no significant differences in adverse events between the vaccine and placebo recipients [59].

The immunogenicity was analyzed in serum specimens collected before immunization and 6 days after the second vaccine dose. Immune responses against LT and O78 LPS (three of the vaccine strains were O78 positive *E. coli*) were determined [59]. However, antibody responses against the CFs were not analyzed because previous studies have shown that such responses are rare in the serum of ETEC-naïve individuals [30,31]. This differs from findings in ETEC-endemic countries such as Bangladesh and Zambia where frequent CF-specific antibody responses against ETVAX were induced in plasma or serum [49,50,52]. Among the Finnish vaccine recipients 81% developed LTB-specific IgA and 73% LTB IgG responses in serum, which were slightly higher for IgA than IgG responses (22- vs. 15-fold) [59]. Responses against O78 LPS were also common (70% IgA) with magnitudes comparable to those previously observed for adult Swedish volunteers [31] as well as children in Bangladesh [51]. Unfortunately, mucosal immune responses could not be determined due to logistical and financial constraints [59].

The secondary objective of the study was to evaluate protection against moderate–severe (MSD) diarrhea associated with ETEC VPO cases without copathogens [60]. However, analyses of diarrheal stool specimens from the MSD VPO cases by real-time PCR [56] revealed that nearly all specimens contained bacterial copathogens, preventing analyses according to the prespecified endpoint. Because of the high frequency of copathogens, i.e., more than 95% of all VPO-positive fecal samples collected from MSD cases contained copathogens, and because unanticipated cross-protection against other enteric bacteria, e.g., *Salmonella*, was observed [60], a revised VPO definition was used, allowing all bacterial copathogens but excluding viral and parasitic copathogens.

Using the revised VPO definition, a vaccine efficacy of 48% (*p* = 0.039) was observed against ETEC-associated MSD cases. A similar efficacy of 48% was also noted against all-cause TD with ≥16 stools per 24 h, indicating disease severity sufficient to prevent daily activities, regardless of ETEC detection [60].

Based on these encouraging results, a clinical trial is planned to evaluate the protective efficacy of ETVAX against ETEC challenge in adult American volunteers.

## 14. Concluding Remarks

The development of an effective ETEC vaccine against enterotoxigenic Escherichia coli (ETEC) disease has been challenging. Based on the success of developing an oral inactivated cholera vaccine [1], it was hypothesized that a similar strategy could be employed for ETEC, given the related pathogenic mechanisms between the two organisms. However, in contrast to *V. cholerae*, which expresses a single O antigen and a single enterotoxin (cholera toxin, CT), pathogenic ETEC strains display greater antigenic and virulence factor diversity. Specifically, ETEC can express at least 80 distinct O antigens, one or more of at least 26 colonization factors (CFs), and one or two enterotoxins.

Identification of the ETEC pathotypes most frequently associated with human disease has therefore been an important step toward vaccine development. Epidemiological data indicate that a limited number of CFs, specifically CFA/I and CS1–CS6, predominate globally and over time, whereas no single O antigen group exhibits comparable prevalence. Moreover, recent studies suggest that approximately two-thirds of ETEC clinical isolates produce heat-labile toxin (LT) alone or in combination with heat-stable toxin (ST).

Based on these insights, and evidence demonstrating that protective immunity in humans is mediated by immune responses against CFs and LT, several vaccine candidates have been developed using inactivated bacteria expressing key CFs in conjunction with LT antigens. Because ETEC infections are confined to the small intestine, a major goal has been to induce potent mucosal immune responses at the intestinal surface, which is best achieved through oral immunization. Based on the studies in animals [3] and the nature of ETEC infections being confined to the small intestine during disease we have previously deduced that the most likely protective mechanisms against ETEC disease are intestinally produced SIgA antibodies against the toxins and CFs in the gut [39]. However, so far no conclusive correlate of protection has been identified in clinical trials.

**Figure 1:**
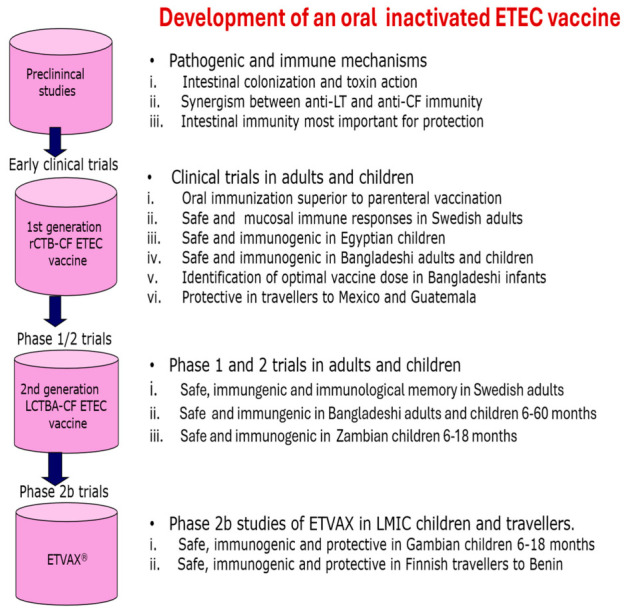
Different steps of oral ETEC vaccine developmen.

The different candidate vaccines described in this review were designed to be safe and to elicit robust immune responses against the key vaccine antigens. The sequential phases involved in the development of an effective ETEC vaccine are summarized in Figure 1.

Preclinical studies in animals were essential for identifying the key protective antigens and determining optimal immunization routes. Based on these results, a first-generation ETEC vaccine was developed and several lots were manufactured by the Swedish company, SBL Vaccin. Clinical trials both in Swedish adult volunteers and in adults and children from various LMICs demonstrated that the different vaccine lots were safe and immunogenic. When tested in American travelers to Mexico and Guatemala, the vaccine was shown to induce significant protection against diarrheal disease that interfered with the travelers’ daily activities, but no protection against mild disease. In a subsequent study of the efficacy of the vaccine in young children in Egypt no significant protection against ETEC-associated diarrhea was observed, most likely due to active surveillance being applied, and hence, most cases were mild.

These results stimulated the development of an improved second-generation ETEC vaccine in collaboration with the Swedish company Scandinavian Biopharma. This formulation employed *E. coli* strains genetically engineered to overexpress CFs and a more LT-like toxoid than used in the first-generation vaccine. The inclusion of a mucosal adjuvant, dmLT, was also evaluated and showed promising immune-potentiating effects.

Multiple clinical studies in both Western adults and in children and adults in LMICs confirmed that the second-generation ETEC vaccine candidates were safe and elicited strong and frequent mucosal immune responses against the CFs and LT. However, frequent serum antibody responses against CFs were only observed in individuals from high endemic regions, likely reflecting priming by natural infection. This finding underscores the importance of assessing mucosal immune responses when evaluating ETEC vaccine immunogenicity, particularly in immunologically naïve populations.

Based on the favorable safety and immunogenicity results in Phase 1 and 2 trials of the second-generation vaccine in Swedish, Bangladeshi and African participants, ETVAX was evaluated for protective efficacy in young children in The Gambia and in travelers to Benin in West Africa. After initial analyses, applying the predefined primary endpoints, no statistically significant protection against diarrhea attributed to ETEC alone or ETEC in combination with a limited number of copathogens was observed for any of the studies. However, subsequent analyses revealed that ≥95% of ETEC-positive MSD stool specimens, collected from both Gambian children as well as Finnish travelers, contained one or more additional enteric pathogens. As a result, the predefined primary endpoint of protection against ETEC-only diarrhea could not be meaningfully assessed due to the low number of ETEC-only cases. When reanalyzed to include MSD cases in which ETEC was detected together with other copathogens, significant protection of 50% was observed in the children in The Gambia and in 48% among travelers.

These findings underscore the importance of conducting efficacy assessments in high-endemicity settings, where protection against the target pathogen should be evaluated in the context of co-circulating enteric pathogens that reflect the typical etiology of diarrheal disease in such environments.

While ETVAX did not provide protection in all Gambian children, the observed vaccine efficacy was comparable to that reported for licensed oral rotavirus vaccines in children in LMICs. A recent meta-analysis of trials of oral rotavirus vaccine conducted in 19 different countries in Latin America, Asia and Africa reported a mean vaccine efficacy of 52% among children aged 6–11 months, which is comparable to the efficacy observed for ETVAX in children aged 6–18 months [61].

ETEC has been reported to cause 75 million diarrhea episodes with up to 42,000 deaths annually in children. Based on the findings in the study in The Gambia that ETVAX provided at least 50% protection against ETEC diarrhea cases in young children and infants and that the vaccine had a coverage of more than 90% of clinical ETEC isolates [54], the vaccine may have the potential to prevent several million ETEC MSD cases and several thousand deaths. In the study in The Gambia it was estimated that the number of children needed to be vaccinated by ETVAX to prevent an episode of MSD ETEC with copathogens was 125 and as few as 50 children to prevent all MSD cases regardless of cause [54].

With regard to the benefits of ETVAX vaccination for travelers, an important finding from the study in Benin is that the vaccination contributed to reducing antibiotic use thereby curbing antibiotic resistance [60].

Taken together the favorable safety profile and encouraging protective efficacy of ETVAX, demonstrated both in African children and Western travelers, provide a strong and well-substantiated rationale for advancing the vaccine to Phase 3 clinical trials.

## Figures and Tables

**Table 1:** Comparison of main virulence factors, putative vaccine antigens in *V. cholerae* and ETEC.

Pathogen	Toxins	O Antigens	Colonization Factors	Additional Putative Vaccine Antigens
*V. cholerae*	Cholera toxin, CT	O1, (O139)	TCP, MSHA *	
Enterotoxigenic *E. coli* (ETEC)	Heat-labile toxin, LT Heat-stable toxin, ST (STh, STp)	>80, e.g., O6, O8, O78, O169	≥26 different, e.g., CFA/I, CS1–CS7, CS17	Eat mucinase, EtpA glyco- Protein etc

* TCP = toxin-coregulated pilus, MSHA = mannose-sensitive hemagglutinin.

**Table 2:** Oral inactivated first- and second-generation ETEC candidate vaccines.

ETEC Candidate Vaccines	Composition
CTB-CF (prototype, first generation)	CTB + inactivated CFA/I and CFA/II positive ETEC clinical isolates
rCTB-CF (first generation)	rCTB + inactivated CFA/I and CS1–CS5 positive ETEC clinical isolates
LCTBA-CF (second generation)	LCTBA+ recombinant ETEC strains overexpressing CFA/I, CS3, CS5, CS6 + LCTBA toxoid ± dmLT
ETVAX^®^ (second generation)	LCTBA + recombinant ETEC strains overexpressing CFA/I, CS3, CS5, CS6 + LCTBA + dmLT

Abbreviations: CTB, cholera toxin B subunit; rCTB, recombinant cholera B subunit; LCTBA, hybrid molecule between rCTB and heat-labile toxin LTB subunit; CF, colonization factor; CFA/I, colonization factor I; CS, coli surface antigen; dmLT, double mutant LT adjuvant.

**Table 3:** Percentage of 25 volunteerswith significant immune responses against CFs and CTB in intestinal lavage, feces, ASCs and serum after two oral doses of rCTB-ETEC vaccine.

Vaccine Antigen	Lavage (%)	Feces (%)	ASC (%)	Serum (%)
CFA/I	87	81	79	56 *
CS1/3	70	52	76	20
CS4	65	43	68	8
CTB	96	90	100	92

* The CFA/I antigen was later shown to contain some O78LPS antigen, explaining the frequent CFA/I response in serum.

**Table 4:** Frequencies of ALS IgA and fecal SIgA antibody responses against the vaccine antigens in Swedish adults given two doses of LCTBA-CF ETEC vaccine, ±10 µg dmLT or placebo.

Antigen	CFA/I	CS3	CS5	CS6	LTB	O78LPS
**ALS responses**	%	%	%	%	%	%
LCTBA-CF vaccine	56	89	56	56	90	100
LCTBA-CF + 10 µg dmLT	71	82	68	71	97	100
Placebo	4	8	4	13	3	0
**Fecal responses**	%	%	%	%	%	%
LCTB-CF vaccine	67	70	70	60	72	70
LCTBA-CF + 10 µg dmLT	58	50	58	54	84	71
Placebo	0	8	4	0	7	6

**Table 5:** Frequencies (%) of ALS IgA or fecal SIgA responses against the vaccine antigens in different age groups of Bangladeshi children receiving two fractionated doses of LCTBA-CF ETEC vaccine (V) or placebo (P).

Antigen	CFA/I		CS3		CS5		CS6		LTB		O78 LPS	
Age Group	V	P	V	P	V	P	V	P	V	P	V	P
24–59 months ALS	97	4	93	10	81	2	81	2	100	6	49	8
12–23 months ALS	74	8	79	5	56	3	51	16	95	12	44	8
6–11 months Feces	51	27	51	27	48	26	47	22	55	30	48	19

## Data Availability

The original contributions presented in this study are included in the article. Further inquiries can be directed to the corresponding author.
